# Ecological Momentary Assessment of Head Motion: Toward Normative Data of Head Stabilization

**DOI:** 10.3389/fnhum.2019.00179

**Published:** 2019-06-04

**Authors:** Peter Hausamann, Martin Daumer, Paul R. MacNeilage, Stefan Glasauer

**Affiliations:** ^1^Chair for Data Processing, Department of Electrical and Computer Engineering, Technical University of Munich, Munich, Germany; ^2^Chair for Computational Neuroscience, Institute for Medical Technology, Brandenburg University of Technology Cottbus-Senftenberg, Senftenberg, Germany; ^3^The Human Motion Institute, Sylvia Lawry Center for Multiple Sclerosis Research e.V., Munich, Germany; ^4^Bernstein Center for Computational Neuroscience, Ludwig-Maximilians-Universität München, Munich, Germany; ^5^Department of Psychology, University of Nevada, Reno, NV, United States

**Keywords:** head stabilization, accelerometry, motion sensors, gait, balance

## Abstract

Head stabilization is fundamental for balance during locomotion but can be impaired in elderly or diseased populations. Previous studies have identified several parameters of head stability with possible diagnostic value in a laboratory setting. Recently, the ecological validity of measures obtained in such controlled contexts has been called into question. The aim of this study was to investigate the ecological validity of previously described parameters of head stabilization in a real-world setting. Ten healthy subjects participated in the study. Head and trunk movements of each subject were recorded with inertial measurement units (IMUs) for a period of at least 10 h. Periods of locomotion were extracted from the measurements and predominant frequencies, root mean squares (RMSs) and bout lengths were estimated. As parameters of head stabilization, attenuation coefficients (ACs), harmonic ratios (HRs), coherences, and phase differences were computed. Predominant frequencies were distributed tightly around 2 Hz and ACs, HRs, and coherences exhibited the highest values in this frequency range. All head stability parameters exhibited characteristics consistent with previous reports, although higher variances were observed. These results suggest that head stabilization is tuned to the 2 Hz fundamental frequency of locomotion and that previously described measures of head stability could generalize to a real-world setting. This is the first study to address the ecological validity of these measures, highlighting the potential use of head stability parameters as diagnostic tools or outcome measures for clinical trials. The low cost and ease of use of the IMU technology used in this study could additionally be of benefit for a clinical application.

## 1. Introduction

During locomotion, reflexive head movements operate to minimize horizontal head translation (Cromwell et al., 2001a; Mazzà et al., 2009) and simultaneously compensate for vertical translation by pitching the head (Pozzo et al., 1990; Hirasaki et al., 1999). These stabilization behaviors are thought to be crucial for effective control of both balance and locomotion because they reduce undesired variability of vestibular and visual sensory inputs (Pozzo et al., 1990). In elderly individuals, head stabilization is compromised during both steady-state walking (Cromwell et al., 2001b) and gait initiation (Laudani et al., 2006; Maslivec et al., 2018). Impaired head stabilization has also been associated with disorders such as Parkinson's disease (PD) (Latt et al., 2009; Buckley et al., 2015), multiple sclerosis (MS) (Psarakis et al., 2018) and bilateral vestibular defects (Pozzo et al., 1991).

Motion capture and accelerometry are widely used in the analysis of head stabilization during human locomotion (Pozzo et al., 1990; Hirasaki et al., 1999; Kavanagh and Menz, 2008). However, studies using motion capture systems are usually constrained to a laboratory setting by design. Similarly, previous studies using wearable sensors have been limited by the need to instruct and supervise subjects and faithfully annotate periods of locomotion. Several recent studies have questioned the ecological validity of measurements obtained in such controlled contexts, i.e., how well these measurements generalize to real world conditions (Stellmann et al., 2015; Brodie et al., 2017). An alternative approach, known as ecological momentary assessment (EMA) (Shiffman et al., 2008), advocates the sampling of clinically relevant parameters in a subject's natural environment rather than a clinical setting.

In support of EMA, researchers have observed that clinical measures such as 10 m walk test times do not significantly correlate with more objective outcomes such as fall risk, raising doubts concerning the clinical relevance of these measures (Brodie et al., 2017). The frequently used 6 min walking test has been challenged by the fact that in many diseased or elderly populations, 6 min of uninterrupted walking rarely occur during daily life (Stellmann et al., 2015). While there are some clinical tests whose results correlate with objective outcomes (such as clinical assessment of gait speed, Albrecht et al., 2001), these examples highlight the need to validate standardized measures in a real-world context.

Wearable accelerometry devices have been suggested for sampling human motion during daily life (Motl et al., 2012) and can be used as a way to assess head stabilization performance in the spirit of EMA. Compared with clinical tests, they provide a cost-effective and straightforward method of recording ecologically valid measures. Previous studies of vestibular stimulation have used these kinds of sensors to address head and whole body motion in more realistic contexts, but were either constrained to pre-defined activities (Carriot et al., 2014, 2017) or lacked measurements of angular velocity (MacDougall, 2005).

In order to assess whether they are indicative of real-life locomotor function, previously established measures of head stability (Hirasaki et al., 1999; Mazzà et al., 2009; Bellanca et al., 2013) need to be evaluated with respect to their ecological validity. Results obtained from a sample of healthy individuals could then be used as a normative baseline for future studies involving populations with balance, gait or neurological disorders.

Therefore, the aims of this study were: (i) to record a dataset of real-world human motion of trunk and head with wearable sensors, (ii) to compute previously described parameters of head stabilization from this data, and (iii) to compare the computed parameters with previous results obtained in controlled environments.

## 2. Materials and Methods

### 2.1. Subjects

A convenience sample of ten healthy human subjects (five male, five female, age 21–28, most of them students participating in lecture “Clinical Applications of Computational Medicine" at the Technical university of Munich) with no history of balance or gait disorders participated in the experiment. All subjects signed an informed consent form compliant with the European General Data Protection Regulation and gave explicit consent to the publication of the recorded data. The study protocol was approved by the institutional review board of the Sylvia Lawry Center for Multiple Sclerosis Research.

### 2.2. Sensor Devices

We used a small, self-contained IMU to record both linear acceleration and angular velocity of the human head and trunk. The device (Actigraph GT9X Link) was chosen for its ability to continuously record accelerometer and gyroscope data at a sampling rate of 100 Hz for 24 h. To record head motion, the sensor unit was firmly attached to the inside of a baseball cap that was worn by the subjects. To record trunk motion, an IMU of the same model was attached to a specialized neoprene belt (actibelt flex-belt, Trium Analysis Online GmbH, Munich, Germany) worn at the waist under the clothing. The actibelt system itself is frequently used in clinical accelerometry studies, but was not used in this study because in its current version it is not equipped with a gyroscope.

### 2.3. Data Acquisition

Subjects were outfitted with the recording equipment in the morning of a typical work/university day and instructed to wear the equipment for at least 10 h. They were instructed to take note of periods during which they took off either sensor unit and these periods were subsequently excluded from analysis. The recording equipment was returned the next morning.

**Figure 1:**
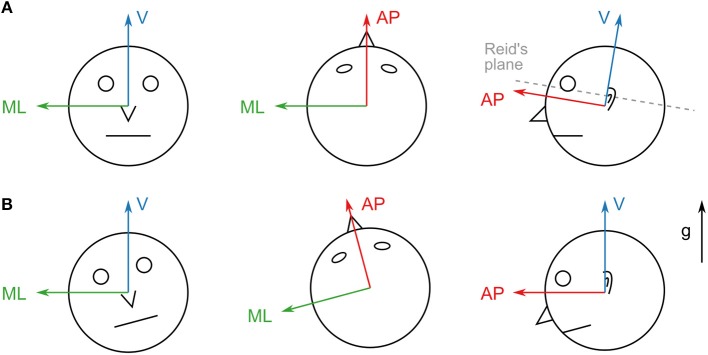
**(A)** Axes of the head sensor coordinate system. The vertical axis is adjusted to be perpendicular to Reid's plane. This coordinate system remains head-fixed during all translations and rotations. **(B)** Axes of the aligned coordinate system. The vertical axis points in the direction of gravity (g). The system remains world-fixed during roll and pitch rotations (left and right) but remains head-fixed during yaw rotations (middle) and translations.

The IMUs were synchronized by knocking both devices against each other at the beginning and the end of each recording. This created clearly visible peaks in the accelerometer measurement that were used to correct timing offsets and drifts between the devices. All subjects performed a calibration routine for both sensor units in order to align the sensor coordinates with head- and trunk-fixed reference frames. For the head device, they first held their heads in a slightly forward-pitched position that aligned Reidâ's plane (MacNeilage and Glasauer, 2017) with an earth-horizontal plane. Afterwards, they nodded their heads five times around the pitch axis. This yields a unique transformation that rotates the acceleration due to gravity to be purely vertical and rotates the angular velocity to be purely around the medial/lateral axis for this calibration routine (resulting in a head-fixed reference frame as shown in Figure 1A). A similar routine was performed for the trunk device which was calibrated such that the acceleration due to gravity was purely vertical when the subjects stood up straight.

### 2.4. Coordinate Frame Transformations

The IMUs used for this study record linear acceleration and angular velocity, but provide no direct information about the orientation of the device in world coordinates. The calibration approach outlined in the previous section yields a head/trunk-fixed coordinate system (Figure 1A). However, for comparability with previously reported results obtained in laboratory settings (Hirasaki et al., 1999; Menz et al., 2003; Mazzà et al., 2009) it is necessary to transform the measurements into a frame of reference whose vertical axis remains parallel to the direction of gravity. Reference frames in these studies are defined as right-handed coordinate systems with the vertical axis pointing upwards in the direction of gravity, the anterior/posterior axis pointing in the direction of the subject's motion and the medial/lateral axis pointing to the left of the motion direction (Figure 1B).

### 2.5. Estimation of the Direction of Gravity From IMU Data

Gravitational acceleration g is linked to linear acceleration a and angular velocity through the following equations (Glasauer, 1992):

(1) $$\begin{array}{r} g=a-i \end{array}$$

(2) $$\begin{array}{r} \frac{\partial g}{\partial t}=\omega \times g \end{array}$$

where *i* denotes the inertial acceleration of the device. Various filters are described in the literature that combine the linear acceleration and angular velocity measurements to produce an estimate of orientation. We propose a basic sensor fusion approach (Table 1) that we show to be sufficiently accurate for typical trajectories occurring during human locomotion.

**Table 1:** Description of the gravity filter algorithm for estimating gravity direction from IMU data.

**Inputs**	Linear acceleration in sensor coordinates *a*(*t*)
	Angular velocity in sensor coordinates ω(*t*)
	Correction factor α
	Acceleration due to gravity in world coordinates *g*_*W*_
**Outputs**	Estimate of acceleration due to gravity in sensor coordinates *g*(*t*)
	Quaternion representing sensor orientation in aligned coordinates *q*(*t*)
**Description**	Start with initial estimate *g*(0) = *g*_*W*_, for all *t* ∈ 1..*T*: 1. Compute estimate of angular displacement: *ϕ*(*t*) = *ω*(*t*)*Δt* 2. Compute estimate of gravity: *g*(*t*) = *ϕ*(*t*) × *g*(*t* − 1) 3. Update estimate with linear acceleration: *g*(*t*) = *g*(*t*) + (1 − α)*a*(*t*) 4. Normalize estimate *g*(*t*) = *g*(*t*)/|*g*(*t*)| 5. Compute *q*(*t*) as the quaternion transforming *g*(*t*) into *g*_*W*_: a. Normalized axis of rotation: *n* = *g*_*W*_ × *g*(*t*), *n* = *n*/|*n*| b. Angle of rotation: $=\cos^{-1}\left(<g_{W},g\left(t\right)>\right)$ c. Quaternion from axis-angle representation: *q*(*t*) = *R*(*n*, θ)

The correction factor α ∈ [0, 1] determines the weight of *ω* in the final estimate; an α of 0 means that *ω* is not used at all, while an α of 1 means that the linear acceleration is ignored. T is the number of samples and Δt is the time difference between two samples, corresponding to 10 ms at a sampling rate of 100 Hz. < g_W_, g(t) > denotes the inner product between g_W_ and g(t) and R(n, θ) computes the quaternion from the axis-angle representation of the rotation:

*R(n, θ) = (cos(θ/2), n_x_sin(θ/2), n_y_sin(θ/2), n_z_sin(θ/2))*.

The angular velocity was high-passed at 0.1 Hz with a 5th-order Butterworth filter to remove errors due to gyroscope drift. The linear acceleration was low-passed with the same type of filter to reduce the influence of transient accelerations on the estimate. The estimates of orientation and acceleration due to gravity from the filter could then be used to transform the raw acceleration measured by the sensor into net inertial acceleration in aligned coordinates:

(3) $$\begin{array}{r} i_{A}=rot\left(q^{-1},a-g\right) \end{array}$$

where *rot*(*q, v*) denotes the rotation of the vector *v* by the quaternion *q*. It should be noted that step 5 of Table 1 ensures that the transformation has no yaw rotation component since *q*(*t*) is computed from a rotation around the axis *n* which is always perpendicular to *g*_*W*_. For consistency with previously reported results (Hirasaki et al., 1999) where translations were described in a world-fixed, but rotations were described in a head/trunk-fixed frame, we did not transform the angular velocity into the aligned coordinate system.

We recorded a short dataset of one subject wearing one IMU attached to a baseball cap on the head. The sensor was mounted facing upwards on a plastic plate equipped with four optical markers for a motion capture system (8 Qualisys Oqus 100 cameras and Qualisys Track Manager software, version 2.9, Qualisys AB, Göteborg, Sweden). The subject performed different locomotor activities (walking, running) as well as spontaneous head movements while sitting for about 8 min. Afterwards, the sensor apparatus was removed from the baseball cap and rapidly swung around, creating high accelerations, and rapid orientation changes of the device for about 1 min. The motion capture data was used as a gold standard for evaluating the accuracy of the orientation estimate as well as finding the optimal parametrization of the algorithm.

We investigated the influence of the low-pass cut-off frequency of the linear acceleration (*f*_*LP*_) as well as the correction factor α on the estimate quality and compared our approach with a previously described complementary filter method (Wetzstein, 2017). The accuracy was measured with the geodesic distance from the estimated quaternion *q* to the gold standard quaternion *q*_*GS*_ (corresponding to the angle of the shortest arc between the two orientations, Huynh, 2009):

(4) $$\begin{array}{r} d=\cos^{-1}\left(2<q,q_{GS}{>}^{2}-1\right) \end{array}$$

Both filter algorithms were implemented in Python 3.6 using the just-in-time compilation tools of the numba library (version 0.42) to greatly enhance execution speed. Run times were compared on an Intel Core i7-7700K CPU in single-threaded execution at a clock rate of 4.2 GHz. Based on the results of this analysis (see Supplementary Material), accelerometer and gyroscope data were transformed to the respective reference frames before further processing.

### 2.6. Step Detection

In order to isolate periods of locomotion for analysis, we used a step detection method based on the inertial acceleration of the trunk sensor in aligned coordinates. We recorded a dataset of one subject wearing the trunk sensor, performing different locomotor activities at different speeds, including walking, running, stair walking, and cycling. This data was used to parametrize a peak detector for extracting possible steps as well as to determine discriminative features that distinguish cycling from other types of motion.

Peaks were detected in the vertical axis component with a minimum height of 0.2 g, prominence of 0.4 g and distance of 20 ms (corresponding to a maximum detectable step frequency of 5 Hz, Schimpl et al., 2011). For each peak, we computed the short-time power spectrum *S*(*f*) of the linear acceleration in all three spatial axes with a segment length of 1,024 samples centered around the peak, weighted with a Blackman window function. The power spectrum was used to determine predominant frequency in each axis, i.e., the frequency with the highest spectral power. We investigated the distribution of RMS vertical accelerations as well as the difference between predominant frequencies in the vertical (V) and medial/lateral (ML) direction and used the results as criteria for the exclusion of cycling periods (see Supplementary Material).

The step detection method was applied to the trunk sensor data for each of the 10 subjects. Since we limited our analysis to frequencies above 1 Hz (see results), detected steps were grouped together as bouts if the time difference between two consecutive steps was smaller than 1 s. Bouts of single steps, i.e., where no other steps where detected within 1 s before and afterwards, were subsequently excluded from further analysis.

### 2.7. Predominant Frequency as a Proxy for Walking Speed

We determined the predominant frequencies of head and trunk accelerations for each step in all three spatial axes using the same short-time power spectrum approach as described above, albeit with a segment length of 512 samples. We used a shorter segment length than in the step detection procedure as it increased the temporal resolution at the expense of frequency resolution, yielding more accurate results for short bouts. We also calculated the magnitude of accelerations using the RMS for each step segment in all three directions. Furthermore, means and standard deviations of trunk predominant frequency in the V direction were calculated for each bout.

In Hirasaki et al. (1999), the authors showed a strong link between walking velocity and predominant frequency of vertical head translation. While we did not validate the exact correspondence for our data, predominant frequency of vertical head acceleration was used as a proxy measure for gait speed, allowing qualitative comparisons between previously published results and ours. In the following, we use the term “predominant frequency” as a shorthand for predominant frequency of vertical head acceleration.

### 2.8. Assessment of Head Stabilization During Locomotion

#### 2.8.1. Attenuation Coefficient

The reduction of linear accelerations through the upper body was quantified for each step segment using the AC between trunk and head. Segments consisted of 512 samples centered around the peak and were weighted using a Blackman window function in order to decrease the influence of non-locomotor accelerations for short bouts. ACs were calculated in the anterior/posterior (AP), ML, and V directions using the RMS values of head (*A*_*H*_) and trunk acceleration (*A*_*T*_) (Mazzà et al., 2009) as:

(5) $$\begin{array}{r} AC=1-\frac{A_{H}}{A_{T}} \end{array}$$

Positive values indicate an attenuation of head accelerations with respect to trunk accelerations whereas negative values correspond to increased accelerations at the head when compared to the trunk.

#### 2.8.2. Harmonic Ratio

Regularity and smoothness of motion was quantified using the HR for both head and trunk accelerations. In the AP and V directions, the HR was calculated as the total spectral power of the even harmonics divided by the total spectral power of the odd harmonics of the predominant frequency:

(6) $$\begin{array}{r} HR=\frac{\sum_{k}^{N}S\left(2kf_{dom}\right)}{\sum_{k}^{N}S\left(2\left(k+1\right)f_{dom}\right)} \end{array}$$

where *f*_*dom*_ denotes the predominant frequency of the segment in the respective direction and *N* = 10 is the number of harmonics we considered. Because of the biphasic nature of accelerations within strides (two steps), high values indicate that acceleration patterns remain in phase across stride cycles and are associated with stable gait (Menz et al., 2003). In the ML direction, the HR was calculated inversely due to the fact that lateral motion is monophasic within one stride (left and right step, Lowry et al., 2012):

(7) $$\begin{array}{r} HR=\frac{\sum_{k}^{N}S\left(2\left(k+1\right)f_{dom}\right)}{\sum_{k}^{N}S\left(2kf_{dom}\right)} \end{array}$$

#### 2.8.3. Coherence

We quantified head-trunk coordination and compensatory head motion during locomotion using the coherence (Hirasaki et al., 1999):

(8) $$\begin{array}{r} K_{xy}^{2}\left(f\right)=\frac{S_{xy}{\left(f\right)}^{2}}{S_{xx}\left(f\right)S_{yy}\left(f\right)} \end{array}$$

where *S*_*xy*_(*f*) denotes the cross-power spectrum of signals *x* and *y*, *S*_*xx*_(*f*) is the power spectrum of signal *x*, and *S*_*yy*_(*f*) is the power spectrum of signal *y*. Coherence values were computed between head pitch velocity and vertical head acceleration and between head pitch velocity and trunk pitch velocity.

As the coherence for the power spectrum of a single segment is ill-defined, we used an extended segment length of 1,024 samples centered around every step. Each segment was divided into 5 sub-segments of 512 samples with an overlap of 128 samples. This approach guaranteed a well-defined coherence measure for each segment with the same frequency resolution as in the rest of the experiments.

#### 2.8.4. Phase Difference

As another measure of head stabilization we used the phase difference between two signals *x* and *y* (Hirasaki et al., 1999). This was calculated by determining the peak of the cross-correlation between *x* and *y*, in segments of 512 samples centered around each detected step. The time-lag of this peak was then transformed into a phase difference by dividing by the period length of signal *x*, estimated via auto-correlation. Phases differences were calculated between vertical head acceleration and head pitch velocity and between vertical head acceleration and trunk pitch velocity.

Since we computed phases differences between acceleration and pitch velocity, we corrected the resulting differences to be comparable with previously reported results that compared vertical displacement and pitch angle (Hirasaki et al., 1999). Pitch angle is obtained from pitch velocity by integrating once (taking into account some initial value) and translation is obtained from acceleration by integrating twice. Since the integration of a sinusoidal signal introduces a phase shift of $-\frac{\pi }{2}$, the overall phase correction for the difference is $2\left(-\frac{\pi }{2}\right)-\left(-\frac{\pi }{2}\right)=-\frac{\pi }{2}$.

### 2.9. Statistical Analysis

The influence of the predominant frequency on the calculated measures was estimated with a Kruskal-Wallis test by calculating an effect size as follows (Tomczak and Tomczak, 2014):

(9) $$\begin{array}{r} \eta^{2}=\frac{H-k+1}{n-k} \end{array}$$

where *H* is the Kruskal-Wallis statistic, *k* is the number of predominant frequency groups and *n* is the number of samples. Effect sizes were considered small for *η*^2^ < 0.04, intermediate for 0.04 < *η*^2^ < 0.11 and large for *η*^2^ > 0.11 (Cohen, 1988). For pairwise comparisons between independent samples (e.g., between previously reported results and ours), Welch's two-sample *t*-test was used. Pairwise comparisons between dependent samples (e.g., between different spatial directions) were performed with a paired *t*-test. For each test, we reported *p*-values and considered results to be significant if *p* < 0.01. However, since this was an exploratory study, statistical power of these tests might be limited.

Statistical analysis was performed with the stats module of the scipy library (version 1.2.0) in Python 3.6. Results of our analyses were plotted as a function of predominant frequency using boxplots. Boxes indicated the range from the first to the third quartile and the band indicated the median. Whiskers were plotted from the lowest sample within 1.5 times the interquartile range (IQR) of the lower quartile to the highest sample within 1.5 times the IQR of the upper quartile. Due to the large amount of samples, outliers were not plotted. The number of samples was *n* = 34455, the number of steps that fell within the analyzed predominant frequency range (93.74% of all detected steps, see results and Supplementary Material).

## 3. Results

**Figure 2:**
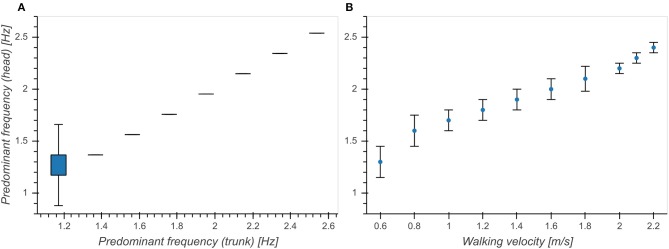
Relationship between predominant frequency of vertical head acceleration and **(A)** predominant frequency of vertical trunk acceleration. **(B)** walking velocity (Figure 8B from Hirasaki et al., 1999). Boxes above 1.2 Hz in **(A)** are not visible because all of the samples between the first and third quartile had the same value.

Predominant frequency of vertical trunk acceleration was strongly correlated with predominant frequency of vertical head acceleration between 1 and 2.6 Hz (*η*^2^ = 0.887, *p* < 0.001, Figure 2A). Figure 2B shows a re-plot of Figure 8B from Hirasaki et al. (1999), showing the relationship between walking velocity and predominant frequency of vertical head acceleration. In order to make our results comparable to previously published results, we limited our analysis to segments with head predominant frequencies between 1 and 2.6 Hz, corresponding to the range of frequencies associated with walking speeds between 0.6 and 2.2 m/s determined in Hirasaki et al. (1999).

**Figure 3:**
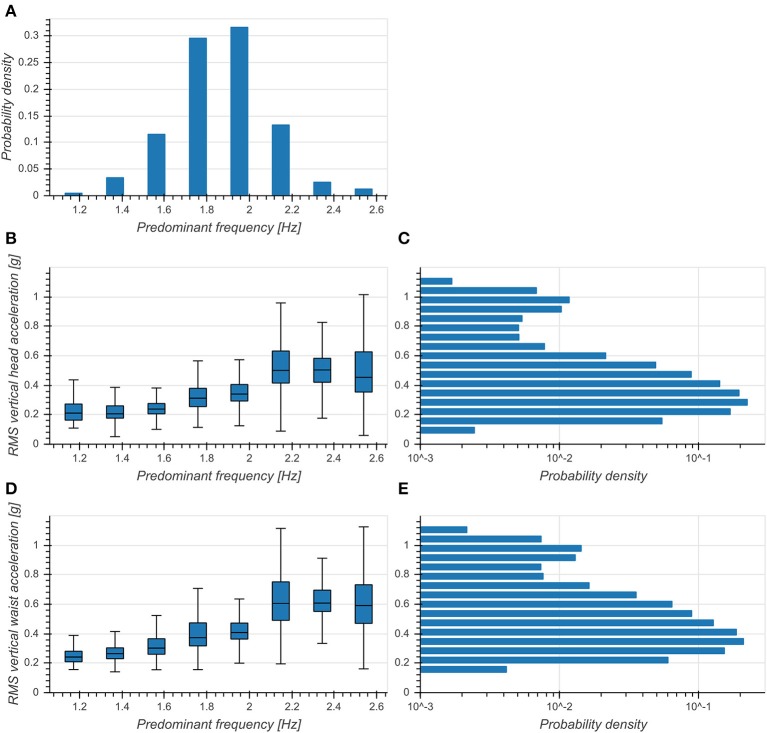
**(A)** Distribution of predominant frequency of vertical head acceleration. **(B)** Boxplot of RMS vertical head accelerations as a function of predominant frequency. **(C)** Distribution of RMS vertical head accelerations (logarithmic scale). **(D)** Boxplot of RMS vertical trunk accelerations as a function of predominant frequency. **(E)** Distribution of RMS vertical trunk accelerations (logarithmic scale).

Predominant frequency of vertical head acceleration was approximately normally distributed around 1.86 Hz with a standard deviation of 0.23 Hz (Figure 3A). RMS vertical accelerations exhibited a distribution skewed toward higher RMS values with a peak at 0.3 g for both head and trunk (Figures 3C,E). RMS accelerations increased with predominant frequency for both head (*η*^2^ = 0.375, *p* < 0.001, Figure 3B) and trunk (*η*^2^ = 0.377, *p* < 0.001, **Figure 5D**) and exhibited broader distributions with higher frequencies. This indicated a strong preference of subjects to move with a fundamental frequency close to 2 Hz and maintaining moderate accelerations of both head and trunk.

**Figure 4:**
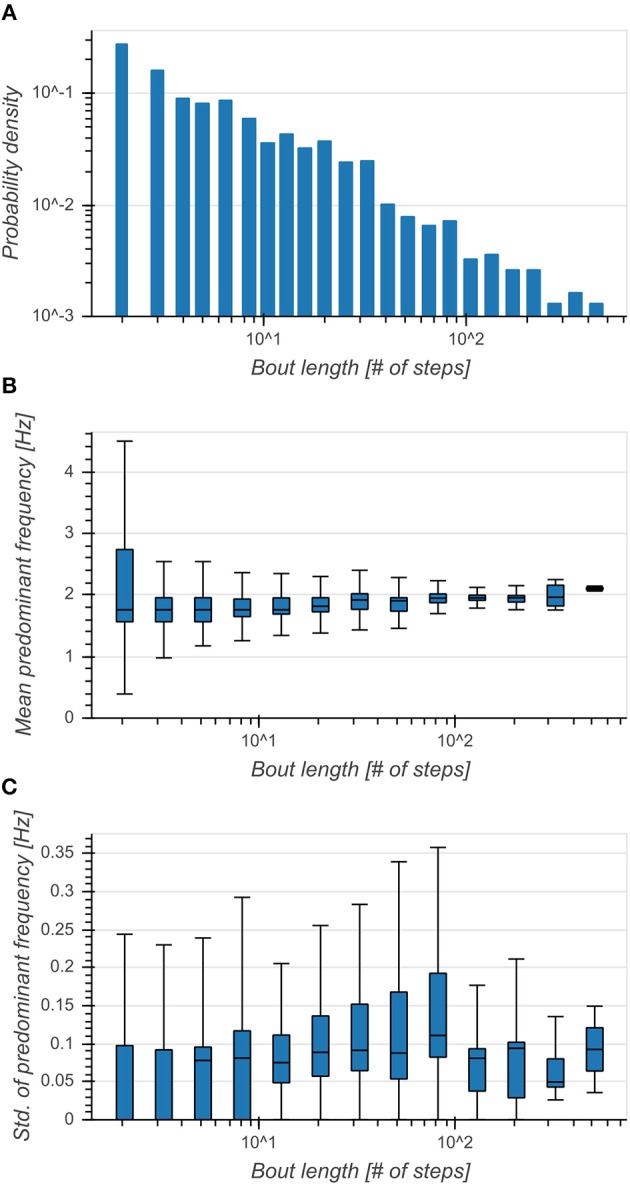
**(A)** Distribution of bout lengths (logarithmic scale). **(B)** Boxplot of mean predominant frequency for each bout as a function of bout length. Broader distributions indicated a higher variance of predominant frequencies between bouts. **(C)** Boxplot of standard deviation of predominant frequency for each bout as a function of bout length. Higher values indicated a higher variance of predominant frequencies within bouts.

Distribution of bout lengths decreased logarithmically with the logarithm of bout length (Figure 4A). The effect of bout length on per-bout mean predominant frequencies was small (*η*^2^ = 0.022, *p* < 0.001), although the median seemed to increase with larger bout lengths and they exhibited broader distributions for shorter bouts (Figure 4B). Standard deviations of predominant frequencies showed an intermediate dependence on bout length (*η*^2^ = 0.101, *p* < 0.001) and exhibited smaller variances above 100 steps (Figure 4C). This showed a clear preference of subjects toward walking short bouts while longer bouts seemed to be connected to an increase of predominant frequency and a simultaneous decrease of variability.

**Figure 5:**
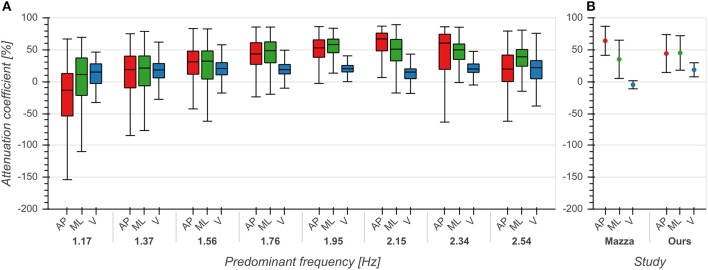
Attenuation coefficients of accelerations between trunk and head in anterior/posterior (AP), medial/lateral (ML) and vertical (V) directions. **(A)** Attenuation coefficients as a function of predominant frequency. **(B)** Comparison between mean +/– std attenuation coefficients from Mazzà et al. (2009) and our data. Means for Mazzà et al. (2009) were computed as the average of the means of the two groups (male, female). Standard deviations were estimated by multiplying the reported standard error of the mean by the square root of the sample size and then computing the square root of the sum of squares of the groups. See also first row of Figure 2 from Mazzà et al. (2009) for comparison.

The effect of predominant frequency on ACs in V direction was small (*η*^2^ = 0.039, *p* < 0.001, Figure 5A). However, ACs increased with predominant frequency up to 2 Hz and afterwards decreased with higher frequencies in both AP (*η*^2^ = 0.165, *p* < 0.001) and ML (*η*^2^ = 0.144, *p* < 0.001) directions (Figure 5A). Pairwise comparisons between directions revealed significant differences between each pair of directions (*p* < 0.001), with ACs in V direction being lower than those in AP and ML directions. These differences were especially evident around 2 Hz, corresponding to the frequency range containing the highest number of samples (see also Figure 3A). ACs in V and AP direction differed significantly (*p* < 0.001) from those reported by Mazzà et al. (2009), but not in the ML (*p* = 0.043) direction (Figure 5B). We found the most substantial difference in the V direction where we observed higher values, indicating that real-world vertical accelerations of the head are more strongly attenuated than previously reported.

**Figure 6:**
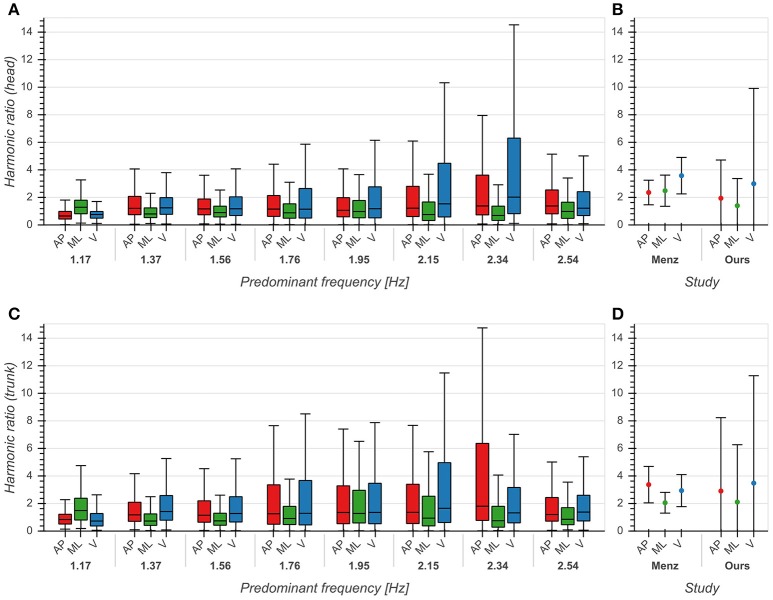
Harmonic ratios of accelerations in anterior/posterior (AP), medial/lateral (ML) and vertical (V) directions. **(A)** Boxplot of harmonic ratios of head accelerations as a function of predominant frequency. **(B)** Comparison between mean ± std harmonic ratios (head) from Menz et al. (2003) and our data. **(C)** Boxplot of harmonic ratios of trunk accelerations as a function of predominant frequency. **(D)** Comparison between mean ± std harmonic ratios (trunk) from Menz et al. (2003) and our data. See also Figure 6 from Menz et al. (2003) for comparison.

The influence of predominant frequency on HRs was small across all directions for both head and trunk (*η*^2^ < 0.04, *p* < 0.001), although we observed higher standard deviations between 2 and 2.4 Hz, especially in the AP and V directions (Figures 6A,C). Distributions differed significantly between each pair of directions (*p* < 0.001). Statistical testing revealed no significant differences between our results and those reported by (Menz et al., 2003) except for the head in the ML direction (*p* < 0.001), but we saw higher standard deviations for all axes and both sensor locations (Figures 6B,D). The high values of HRs measured around 2 Hz are an indication of highly regular and stable gait in this frequency range.

**Figure 7:**
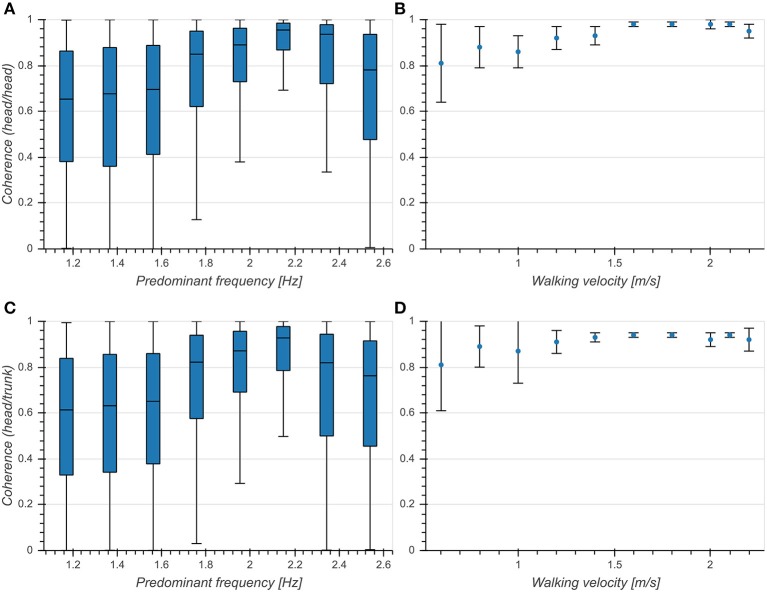
Coherence at predominant frequency. **(A)** Boxplot of coherence between vertical head acceleration and head pitch velocity as a function of predominant frequency. **(B)** Coherence between vertical head displacement and head pitch angle as a function of walking velocity [Figure 9A from Hirasaki et al. (1999)]. **(C)** Boxplot of coherence between head pitch velocity and trunk pitch velocity as a function of predominant frequency. **(D)** Coherence between head pitch angle and trunk pitch angle as a function of walking velocity [Figure 9B from Hirasaki et al. (1999)] .

There was an intermediate effect of predominant frequency on coherence both between vertical head acceleration and head pitch velocity (*η*^2^ = 0.109, *p* < 0.001, Figure 7A) and between head and trunk pitch velocity (*η*^2^ = 0.084, *p* < 0.001, Figure 7C). We observed an increase of mean coherence value around 2.15 Hz as well as a decrease of standard deviation. Coherence values differed significantly between head and trunk in the predominant frequency range from 1.37 to 2.34 Hz. These results are consistent with those reported in Hirasaki et al. (1999) (Figures 7B,D), although it should be noted that they obtained values for vertical displacement and pitch angle instead of vertical acceleration and pitch velocity. However, since the coherence measures the similarity between signals at the predominant frequency, a mere phase shift as introduced by the integration of a sinusoidal signal component should not alter the value of the coherence function. These results demonstrate a tight coupling between both head pitch and vertical head translation as well as head and trunk pitch around the preferred predominant frequency of 2 Hz.

**Figure 8:**
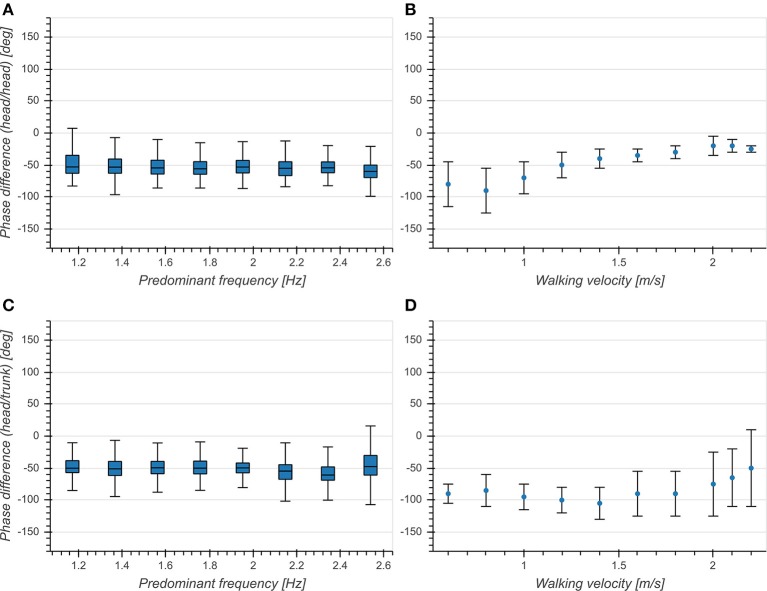
**(A)** Boxplot of corrected phase difference between vertical head acceleration and head pitch velocity as a function of predominant frequency. **(B)** Phase difference between vertical head displacement and head pitch angle as a function of walking velocity [Figure 5C from Hirasaki et al. (1999)]. **(C)** Boxplot of corrected phase difference between vertical head acceleration and trunk pitch velocity as a function of predominant frequency. **(D)** Phase difference between vertical head displacement and trunk pitch angle as a function of walking velocity [Figure 5B from Hirasaki et al. (1999)].

Predominant frequency had a small effect on phase differences for both head (*η*^2^ = 0.006, *p* < 0.001, Figure 8A) and trunk (*η*^2^ = 0.022, *p* < 0.001, Figure 8C). There was a significant difference between head and trunk for the whole analyzed range of predominant frequencies except for 1.17, 1.37, and 2.15 Hz. While the overall mean phase differences were comparable to those reported in Hirasaki et al. (1999), we did not observe a dependence on predominant frequency (Figures 8B,D). This indicates a phase lock between vertical head displacement and head/trunk pitch angle, independent of predominant frequency.

## 4. Discussion

Due to the limited ecological validity of measurements obtained in a controlled laboratory setting (Motl et al., 2012; Brodie et al., 2017), there is a need for methods to measure and analyze head stabilization and head-trunk coordination in real-world scenarios. For clinical applications, it is first necessary to obtain normative data from healthy individuals as a baseline for possible diagnostic use. In this study, we measured head and trunk motion in an ecologically valid context and calculated several derivative measures of head stabilization performance. These measures were chosen based on those reported in the literature, and they evaluate horizontal head stabilization as well as head motion that compensates for vertical translation. Overall, our measures based on real-world accelerometry data agree quite well with similar measures derived from laboratory-based data, suggesting that these methods for quantifying head stabilization performance could generalize. However, we noticed some important differences and in general we observed larger variances in the distribution of these measures.

Predominant frequencies of motion were tightly coupled between trunk and head (Figure 2) and exhibited a narrow distribution around 2 Hz (Figure 3). Incidence of bout lengths decreased strongly toward longer bouts, but means and standard deviations of predominant frequencies did not strongly depend on bout length, showing only a small increase of means and simultaneous decrease of standard deviations toward longer bouts (Figure 4). These findings seem to confirm previous reports (MacDougall, 2005) which identified 2 Hz as the fundamental frequency of human locomotion across a wide range of activities. The observed changes in predominant frequency distribution as a function of bout length indicate a tendency of subjects toward more goal-directed and stable walking for longer distances. However, the observed differences for short bouts could also have other causes: On the one hand, these bouts could consist of false positive steps detected during cycling. With a larger annotated dataset it should be possible to develop a more refined step detection approach, possibly involving machine learning techniques or GPS data. Special care needs to be taken in order to faithfully detect slow or asymmetric gaits if the goal is to develop a diagnostic tool. On the other hand, it is possible that this in an artifact of the spectral analysis used for determining predominant frequency, which analyses segments of 5 s length in order to achieve the desirable frequency resolution. This choice arguably influenced the analysis of very short bouts as non-locomotion data was included in the transform window. Yet, for the analysis of elderly people and pathological gaits, short bouts are of paramount importance, as they make up most of the daily walking activity (Schimpl et al., 2011). Special frequency analysis techniques for non-stationary data such as the empirical mode decomposition Huang et al., 1998 could help circumvent this issue.

Attenuation of accelerations from trunk to head was stronger in AP and ML directions than in the V direction (Figure 5), consistent with previous reports (Kavanagh et al., 2005; Mazzà et al., 2009). The reason for this is that the kinematic chain of the upper body aims at minimizing horizontal accelerations in order to stabilize the head in space. Compared with the results of Mazzà et al. (2009) we observed stronger attenuation in the V direction; this could be due to characteristics of our uncontrolled environment such as inclusion of stair walking. Buckley et al. (2015) observed that attenuation of accelerations in the ML direction was significantly lower in patients with Parkinson's disease when compared with healthy controls. This deterioration in patients seems to indicate that attenuation of lateral accelerations is due to active stabilization and not simply biomechanical constraints of the head-trunk chain. Attenuation strengths in AP and ML directions also showed a dependence on predominant frequency, exhibiting the highest values around 2 Hz. To the best of our knowledge, this is the first time that ACs were characterized as a function of predominant frequency. These results suggest that the attenuation of horizontal head accelerations is tuned to the fundamental frequency of locomotion and that the quantification of this attenuation could be used as an ecologically valid objective measure of head stability.

Regularity of motion as measured by the HR was consistent with previous reports (Menz et al., 2003), although we found higher variances in all directions of motion (Figure 6). This could be explained by the fact that a significant effect of environmental factors such as walking on uneven surfaces (Menz et al., 2003) or unilateral limb loading (Bellanca et al., 2013) on the measured HRs has been observed. Previous studies found significantly lower HRs at both trunk and head between patients with MS (Psarakis et al., 2018) or PD (Latt et al., 2009; Lowry et al., 2009) and healthy controls, although there have been differing reports in the case of PD (Buckley et al., 2015). We observed an increase in HRs with predominant frequencies above 2 Hz, most prominently in the AP and V directions, in accordance with earlier reports (Menz et al., 2003). Based on these findings, we conclude that the HR might be a suitable measure of head stabilization in a real-world context.

The similarity between vertical head acceleration and head pitch and between head pitch and trunk pitch as measured by the coherence was maximal at predominant frequencies around 2.2 Hz (Figure 7). This is in line with previous reports (Hirasaki et al., 1999) which observed the highest coherence values at walking speeds above the most common gait velocity of 1.4 m/s. Compared to their results, we measured lower means and higher standard deviations of coherence values across the entire range of analyzed predominant frequencies. These differences can be explained by the fact that Hirasaki et al., 1999 analyzed steady-state walking on a treadmill with a target for gaze fixation. High coherences are associated with compensatory head motion aimed at maintaining gaze stability (Hirasaki et al., 1999). In a real-world setting, often characterized by intermittent walking and frequent gaze shifts, it is not surprising that overall lower coherence values are observed. Lower coherences have also been linked to vestibular deficits (Pozzo et al., 1991), suggesting a possible applicability of this measure in a clinical context.

Phase differences between vertical head acceleration and head/trunk pitch were distributed around −50° across the entire analyzed range of predominant frequencies (Figure 8). This is partly consistent with previous studies (Hirasaki et al., 1999), however these studies reported an effect of walking velocity on the phase difference which we did not observe. Similar to the coherence, we hypothesize that the observed differences are due to our measurement scenario lacking a target for gaze fixation. We are not aware of any studies investigating phase differences of subjects with gait, balance or neurological disorders.

With the exception of phase differences, all analyzed metrics indicated strongest head stabilization around 2 Hz, corresponding to the preferred walking speed of the participants. We also observed the lowest variances of these measures in this range, in line with previous reports by Wuehr et al. (2013) who showed that coefficients of variation of gait parameters such as stride time and stride length are lowest at self-selected walking speeds. Additionally, they measured higher variances in patients with cerebellar ataxia, especially outside of the range of preferred speeds, raising the question whether similar effects could occur for parameters of head stability.

Another disorder characterized by movement deficits is autism spectrum disorder (ASD) (Trevarthen and Delafield-Butt, 2013). Children diagnosed with ASD exhibit atypical motor patterns that can be identified using machine learning techniques with great accuracy (Anzulewicz et al., 2016). Computer-vision based tracking of head motion revealed that magnitude and velocity of head turning as well as velocity of head inclination are greater in children with ASD than in healthy controls (Cassell et al., 2018). This difference was especially evident when subjects watched video of social stimuli. Therefore, assessment of head motion during real-world social interactions could be a valuable tool for ASD diagnosis and research.

It should be noted that the size and makeup of our sample of participants is a possible source for bias. The sample included exclusively young subjects which facilitated comparison with previously reported results. In contrast, a normative dataset for comparison with diseased populations will likely have to include older subjects. A longer measurement period (at least one week) could also be helpful in increasing the significance of findings. Furthermore, neither the gravity estimation nor the step detection algorithm have been independently validated and we did not control for movement of the sensors relative to head or trunk. However, all analyses performed in the aligned coordinate system are largely robust to small shifts in sensor position. The other concerns can be addressed in the study design of future studies.

In conclusion, we have shown that several previously described head stability parameters, when measured in an ecologically valid context, exhibited characteristics similar to those obtained in a laboratory setting. We have also characterized these parameters in function of predominant frequency as a proxy for walking speed (Figures 5–8). Nevertheless, we found some critical differences that could be attributed to features unique to the real-world context. Real-world measurements of attenuation coefficients were comparable to those previously obtained in a laboratory setting (Mazzà et al., 2009), as were measurements of harmonic ratios (Menz et al., 2003). We could also replicate previously reported characteristics of coherences and phase differences (Hirasaki et al., 1999). Most of these measures have been shown to have value for diagnostic purposes or as endpoints for clinical trials. Our results indicate that the evaluated parameters are largely robust to characteristics that are usually absent in a laboratory context, such as frequent and large shifts of gaze and attention, dual tasking or walking with a companion. The data recorded in this study could serve as a model for collecting normative reference data of healthy individuals. Future studies will have to address the direct comparison of ecologically valid head stabilization parameters between healthy controls and patients with gait, balance, or neurological disorders. This way, mobile accelerometry could serve as a cheap and easy method to gain clinically relevant insights.

## Ethics Statement

This study was carried out in accordance with the recommendations of the institutional review board of the Sylvia Lawry Center for Multiple Sclerosis Research in accordance with written informed consent from all subjects. All subjects gave written informed consent in accordance with the Declaration of Helsinki and the European General Data Protection Regulation. The protocol was approved by the institutional review board of the Sylvia Lawry Center for Multiple Sclerosis Research.

## Author Contributions

PH, MD, PM, and SG conceived and designed the experiments, contributed materials and analysis tools, wrote the paper and developed algorithms. PH performed the experiments and analyzed the data.

### Conflict of Interest Statement

MD is the Director of the Sylvia Lawry Center for MS Research. He is managing director of Trium Analysis Online GmbH (50 % ownership). Trium is a manufacturer of CTG monitoring systems. He is an Academic Editor for PeerJ and has invented the “free heel running pad.”

MD has served on the scientific advisory board for the EPOSA study; has received funding for travel from ECTRIMS; serves on the editorial board of MedNous; is co-author with Michael Scholz on patents re: Apparatus for measuring activity (Trium Analysis Online GmbH), method and device for detecting a movement pattern (Trium Analysis Online GmbH), device and method to measure the activity of a person (Trium Analysis Online GmbH), co-author with Christian Lederer of device and method to determine the fetal heart rate from ultrasound signals (Trium Analysis Online GmbH), author of method and device for detecting drifts, jumps and/or outliers of measurement values, coauthor of patent applications with Michael Scholz of device and method to determine the global alarm state of a patient monitoring system, method of communication of units in a patient monitoring system, and system and method for patient monitoring; serves as a consultant for University of Oxford, Imperial College London, University of Southampton, Charité Berlin, University of Vienna, Greencoat Ltd, Biopartners, Biogen Idec, Bayer Schering Pharma, Roche, and Novartis; and receives/has received research support from the EU-FP7, BMBF, BWiMi, and Hertie Foundation.

PH is an employee of the Sylvia Lawry Centre for MS Research.

The remaining authors declare that the research was conducted in the absence of any commercial or financial relationships that could be construed as a potential conflict of interest.
