# Supplementary material for: Ecological Momentary Assessment of Head Motion: Toward Normative Data of Head Stabilization
Source: Front Hum Neurosci. 2019 Jun 4;13:179. doi: 10.3389/fnhum.2019.00179 (PMC6558158; doi:10.3389/fnhum.2019.00179)
Supplement: Supplementary file 1 [file Data_Sheet_1.PDF]

frontiers

Supplementary Material

1 SUPPLEMENTARY DATA

1.1 Estimation of the direction of gravity from IMU data

For the locomotion data, the proposed filter approach - termed gravity filter (GF) - showed a similar performance to the complementary filter (CF), with a minimum root mean square (RMS) orientation error of 7.2° at a low-pass cut-off frequency of $f_{LP} = 0.75$ Hz and a correction factor of $\alpha = 0.1$. The influence of α on the gravity filter's performance was small, although higher factors resulted in higher errors. The choice of f_{LP} had a strong effect; errors increased with higher cut-off frequencies up to 9° at 5 Hz (fig. S1A and B). For the high acceleration data, errors were significantly higher, in the case of the GF with a minimum of 51.5° at $f_{LP} = 1$ Hz and $\alpha = 0.8$. Errors decreased with higher values of and exhibited a flat minimum for values of f_{LP} between 1 and 2 Hz for both GF and CF (fig. S1C and D). A notable exception is the case of $\alpha = 0.9$ that resulted in higher overall errors for the GF but lower errors for the CF, even with increasing f_{LP}. Regarding execution speed, the GF implementation outperformed the CF implementation by a factor of 4, requiring about 3 s per hour of recording compared with 12 s in the case of the CF.

As a result, we used the GF with $f_{LP} = 1$ Hz and $\alpha = 0.8$ to transform the raw accelerometer and gyroscope data before further analysis. This parametrization provided a trade-off between the two motion scenarios in our test data. It should be noted that high acceleration scenario contains data that is very unlikely to occur during natural human locomotion and the corresponding high orientation errors are not a cause for concern.

1.2 Step detection

The relationship between RMS vertical trunk acceleration $A_{T,V}$ and the difference between predominant frequencies in in vertical (V) and medial/lateral (ML) direction Δf_{dom} for the different activities in the dataset is shown in figure S2. Peaks detected during cycling were clustered around $\Delta f_{dom} =$ -2.5 Hz and $A_{T,V} = 0.2$ g. Most locomotion segments exhibited a non-negative Δf_{dom} except for some stair walking data which in turn showed values of $A_{T,V}$ greater than 0.4 g. On the basis of these results we classified all segments with both $\Delta f_{dom} < 0$ Hz and $A_{T,V} < 0.4$ g as cycling and excluded them from further analysis. This rule eliminated over 80 % of incorrectly identified steps in the test dataset while rejecting less than 2.5 % of actual walking and running steps. Since the number of peaks detected during cycling was low to begin with, this approach detected only 52 steps in a 5 minute period of cycling, less than 2 % of the number of steps occurring during a walking bout of the same length. In the analyzed dataset, we detected a total of 36755 steps.

2 SUPPLEMENTARY TABLES AND FIGURES

2.1 Figures

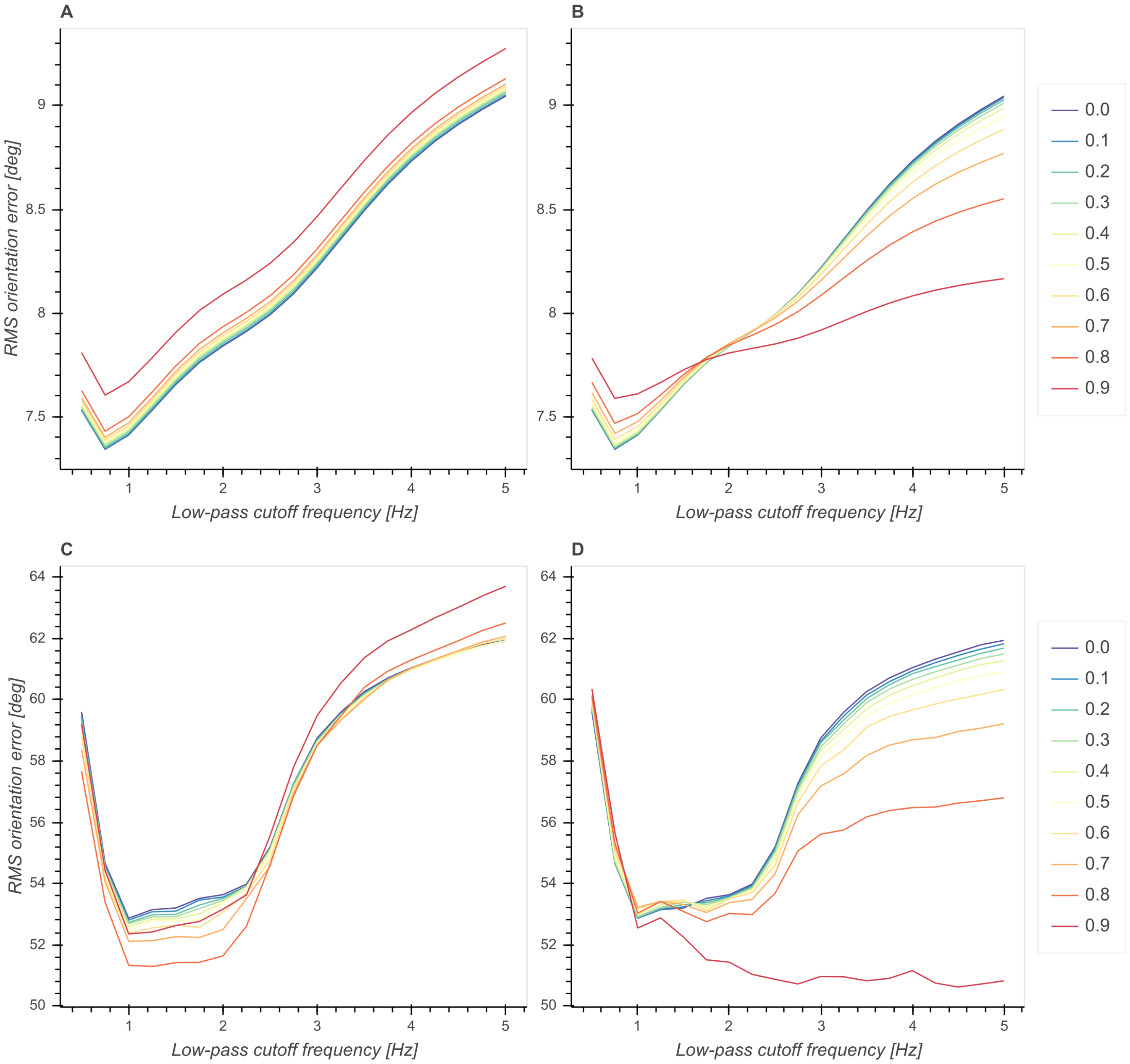

Figure S1. Effect of low-pass cut-off frequency f_{LP} and correction factor α (colored lines) on RMS orientation error of **(A)** gravity filter estimate for locomotion data; **(B)** complementary filter estimate for locomotion data; **(C)** gravity filter estimate for high acceleration data; **(D)** complementary filter estimate for high acceleration data

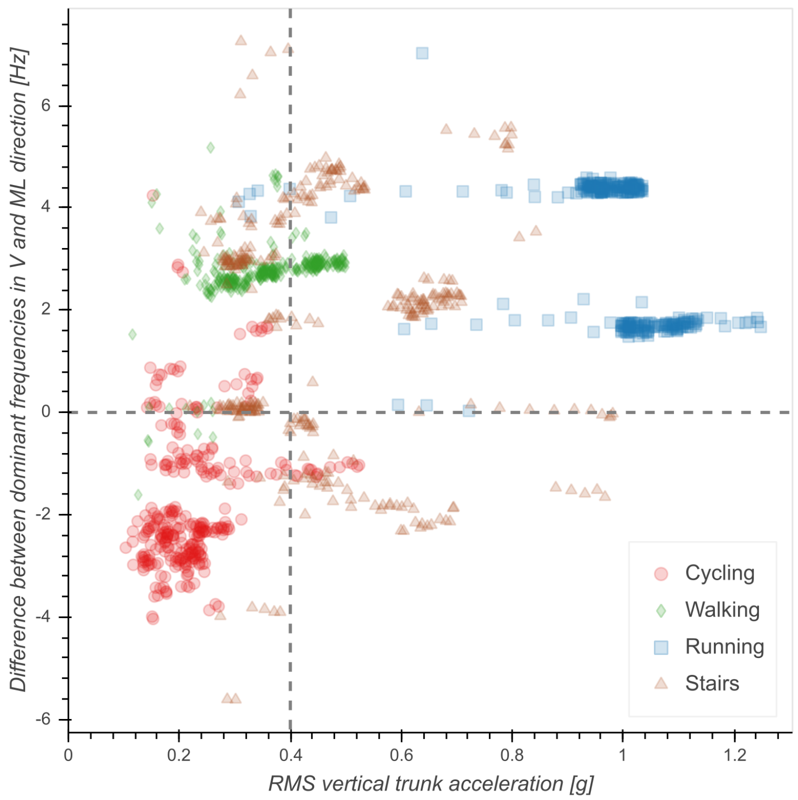

Figure S2. Relationship between difference of dominant frequency Δf_{dom} and RMS vertical trunk acceleration $A_{T,V}$ for the four types of annotated activities (cycling, walking, running, stair walking). A small random offset was added to each value of Δf_{dom} in order to make clusters in the data easier to distinguish.
